# Assessing the training needs of medical students in patient information gathering

**DOI:** 10.1186/s12909-020-1975-2

**Published:** 2020-03-02

**Authors:** Conor Gilligan, Sonja P. Brubacher, Martine B. Powell

**Affiliations:** 1 School of Medicine and Public Health, University of Newcastle, University Drive, Callaghan, NSW 2308 Australia; 2 Centre for Investigative Interviewing, Griffith Criminology Institute, Griffith University, Mount Gravatt, Australia

**Keywords:** Communication skills, Medical interviewing, Faculty development

## Abstract

**Background:**

Effective communication is at the heart of good medical practice but rates of error, patient complaints, and poor clinician job satisfaction are suggestive of room for improvement in this component of medical practice and education.

**Methods:**

We conducted semi-structured interviews with experienced clinicians (*n* = 19) and medical students (*n* = 20) to explore their experiences associated with teaching and learning clinical communication skills and identify targets for improvements to addressing these skills in medical curricula.

**Results:**

Interviews were thematically analysed and four key themes emerged; the importance of experience, the value of role-models, the structure of a consultation, and confidence.

**Conclusions:**

The findings reinforce the need for improvement in teaching and learning communication skills in medicine, with particular opportunity to target approaches to teaching foundational skills which can establish a strong grounding before moving into more complex situations, thus preparing students for the flexibility required in medical interviewing. A second area of opportunity and need is in the engagement and training of clinicians as mentors and teachers, with the findings from both groups indicating that preparation for teaching and feedback is lacking. Medical programs can improve their teaching of communication skills and could learn from other fields s to identify applicable innovative approaches.

## Background

Effective communication is at the heart of good medical practice. Doctor patient relationships, and consequently patient outcomes, are developed through patient-centred, empathetic communication [1]. There is mounting evidence for the influence of doctor-patient communication on a range of measurable patient outcomes [2–5]., and on the flipside, problems with communication are implicated in a large proportion of medical errors, with evidence that failures in the process of taking a medical history from a patient are responsible for a substantial proportion of diagnostic errors in primary care [6–9]. Even in surgery, only 4.3% of errors are associated with the surgery itself; far more are linked to decision making and communication [10–12].

Traditionally, communication has been regarded as one of the ‘soft’ or non-technical skills of medical practice [13] and has been taught separately from the medical science and physical examination skills seen as central to training. Increasingly, however, there is recognition of the complexity of communication skills and importance of integrating these skills as a core component of training [14, 15]. Such a shift has been assisted by the World Federation for Medical Education, with the inclusion in their 2015 Basic Medical Education Standards [16] of communication as a core clinical skill, recommended to be taught as part of early patient contact. International medical curricula accrediting bodies have followed suit, with emphasis on communication as a core curriculum element [17–19].

Despite this recognition of importance, effective approaches to teaching communication skills are less well known. Hundreds of educational interventions are being implemented in medical curricula and professional training programs internationally, with many evaluated in research trials. The evidence, however, remains limited by the theoretical basis of the interventions [20, 21], the evaluation methodologies used, and the feasibility of assessing long-term impacts on practitioner behaviours and student outcomes [22, 23]. Many assessment methods focus on the information obtained or content covered in medical interviews or consultations, with less scrutiny of the processes used to obtain this information. This tension between process and content has long been recognised as a challenge in medical education; both components need to be addressed but are often taught separately [24]. Ideally, communication skills teaching should include some focus on the content – the medical information sought in a consultation. Equally, teaching about medical content and diagnostic reasoning should also include some focus on the process - the communication skills used to obtain information. In the present paper, our references to communication skills are intended to include both the process and content elements of the medical consultation.

The available evidence supports experiential learning models, with the use of actors as simulated patients, and opportunities for feedback and practice [25–27]. Multitudes of studies have shown short-term effects of such approaches on specific learning outcome measures. For example, interventions targeting counselling for changing unhealthy habits can improve students’ adherence to recommended protocols [28, 29]. Likewise, those focused on breaking bad news or other specific content areas improve students’ adherence to recommended approaches and skills in dealing with related scenarios [30–32]. Communication with patients in clinical settings is varied however, and dealing with this variability requires flexibility on the part of the clinician. There is less evidence in the existing literature for how students can be prepared for the flexibility required in clinical communication.

The skills traditionally regarded as most complex in medicine are those psychomotor skills that require well developed hand-eye coordination and dexterity such as surgical skills [10]. There is a plethora of literature on approaches to teaching such skills, with recommendations for breaking the skill down into its component parts, correcting errors through immediate feedback, and using multiple, short practice opportunities [33]. While these steps also form the basis of much communication skills training, they may not be adequate for dealing with the complexity and variability of communication. Unlike a surgical procedure which has a defined protocol and is a generally predictable set of steps to be followed in each case, communication varies from patient to patient even in almost identical clinical situations. The psychosocial, personality, language, and cognitive elements associated with communication make this challenge unique.

Many medical schools use classroom role-play simulation experiences in advance of students practicing skills with real patients but it is not clear how much of what is taught in the classroom is implemented in clinical placement [34, 35]. Students cite difficulty understanding what is expected in different contexts, and feeling pressure from supervisors and time constraints to focus on the medical content, with less time for rapport-building and other patient-centred aspects of the consultation [35]. Further, the communication modelled by faculty in classroom settings is viewed as different to that modelled in the clinical setting [34, 35]. This is likely to create confusion, and leads one to question the degree to which classroom sessions prepare students for clinical experience. The role-modelling observed in clinical practice acts as a hidden curriculum, with powerful influence upon students’ development [36, 37].

While there are many well established methods for teaching communication skills, the degree to which they are perceived to be effective by students and clinicians is less clear. It is important to understand the value of these teaching and learning methods from both the perspectives of those who teach, and those who learn, as a lack of congruence between students’ and teachers’ perspectives can be a barrier to learning [36, 38].

### Aim

This study explored the experiences and perspectives of both experienced clinicians and medical students of learning skills for communicating with patients to gather information in medical interviews. The goal was to identify gaps in the preparation of students and areas for potential improvement to medical curricula.

## Methods

The data presented here were part of a larger study designed to identify parallels between the communication skills used in medical and investigative interviewing. In this context investigative interviewing relates to interviewing in a forensic context, which can include interviewing vulnerable and other witnesses to crime. Therefore, the language used in interviews was ‘information gathering’ as that was the element expected to be most common between the two fields. Here, we focus on data relating to the training needs of medical students. Insights from the experienced clinicians that parallel communication in investigative interviewing can be found in Brubacher et al. ([39] in press).

### Participants

Participants were invited to participate in voluntary semi-structured interviews on the topic of information gathering in medicine. Thirty-two medical students from a large Australian university were invited to participate using convenience sampling, through word of mouth (i.e., an email invitation sent via lecturers and other students), and twelve declined or were unavailable. Students were selected to represent a range of year and experience levels and achieve a heterogeneous sample. Twenty students (11 females, 9 males) participated, including five first-, five second-, two third- and eight fourth-year students. They were aged between 21 and 67, with an average age of 31.55 years (standard deviation = 11.49). All participants noted their English proficiency as ‘fluent’. Students were recruited beyond data saturation to ensure a relatively even sampling of students and clinicians. The medical program in which the students were enrolled was a four-year postgraduate program, with the first 2 years largely occurring on the university campus and the second 2 years occurring exclusively in clinical settings. Classroom ‘communication skills’ sessions begin in the first 2 years and are complemented by both simulated and real patient experience later in the program.

Clinicians were recruited using a combination of snowball and convenience sampling to ensure representation of a wide range of clinical specialties. The criteria on which participants were identified and invited were that they were involved in both teaching or clinical supervision of medical students as well as clinical practice, and were regarded as good communicators. Nineteen medical professionals (10 females, 9 males) with more than 6 years of experience (*M* = 18 years, range = 6–36 years) in their respective area were contacted and nineteen agreed to take part (one declined due to other commitments). Recruitment and data collection continued until saturation was reached, and the researchers were confident that key themes were consistent across different clinical disciplines. Participants represented a broad range of clinical disciplines: cardiology, endocrinology, family medicine, gastroenterology, geriatrics, hepatology, neurology, nursing, obstetrics, pharmacology, general surgery, paramedicine, and psychiatry.

Two of the study authors individually conducted interviews with participants, at a convenient time in a quiet area of participants’ place of study or work (*n* = 37), or on the phone (*n* = 2), with an average duration of 46 min. All participants gave written informed consent. The study was approved by the university’s human research ethics board.

### Materials and procedure

Interviews began with an invitation for participants to give an overview of their background, followed by a question about the role of communication in medical interviews in their specialty area, and a question about challenges related to medical interviewing. Interviewers prompted for more elaboration if responses were brief or unclear but did not deviate from the topics. See additional file 1 for a copy of the interview guide which was developed for this study.

#### Coding

All **i**nterviews were audio recorded, transcribed, and de-identified. Interview transcripts were read separately by three of the study authors (one who had conducted some interviews and two who had not). Thematic analysis was employed to identify the themes using both theory-driven and grounded-theory approaches [40]. We expected to identify themes that would bear relation to training communication skills such as the role of practice opportunities, but we allowed our lists to grow when unexpected themes emerged. Themes were identified by grouping all concepts that arose, regardless of how many raised them, under overarching themes. Once each author had identified themes to exhaustion, they met to discuss the data, and agreed on the same overarching themes. The data were rich with themes and concepts concerning multiple aspects of medical communication. Here, we explore themes relating to the training of medical students, from both students’ and clinicians’ perspectives.

## Results

Four overarching themes emerged which relate to the training of medical students; the importance of experience, the value of role-models, the structure of a consultation, and confidence. Within the first two themes, sub-themes were identified as described below. Quotes from experienced clinicians (experts) are labelled as E [participant] and those from students are labelled as S [participant].

### Learning from experience

Both students (*n* = 9) and experienced clinicians (*n* = 11) reflected on the value of experience in developing their skills, and particularly, knowing what questions to ask and what areas to explore. Clinicians credited the development of their techniques and confidence to the extensive experience they had gained over thousands of patient consultations.

“Unless you’ve literally spoken to hundreds of people with this problem, your radar for picking up these subtler things isn’t there.” [E5].

“I see something or I feel something that’s wrong. It’s the experience, you pick it up. There’s no training, there’s no chapter in the book [that compares].” [E11].

Also, most clinicians stated that they had very little teaching of communication skills during their own training, so they predominantly learnt ‘on the job’.

“We just do what we do and then we try and interpret it as to what strategy were you trying to use. Like you just learn because you’re doing something … I went through medicine in an era where there’s none of this ‘how do you talk to people properly’ this is a new phenomenon.” [E13].

“No mock training, just learning on the job. We did no role playing when I was in medical school.” [E8].

Students saw their lack of experience as a weakness and aspired to the confidence they saw in ‘good communicators’ who had developed their skills over many consultations.

“[What’s important] is me feeling like I’m doing it in a professional manner, and I understand that that comes with experience, that as you learn the key features to look for and you’re doing the sort of diagnosis in your head as you’re going along, you’ll get better and better at it.” [S4].

“Inexperience [is my weakness]. You see a good consultant do it and they make it look really easy, and then you try and do it and it’s really not easy at all.” [S11].

#### Learning from practice opportunities

Five of the clinicians and fourteen students highlighted the role of practice in their acquisition of good communication skills, with clinicians in particular mentioning the importance of “repetition” [E10 and E15]. Clinicians also reflected on the learning process as one involving making and learning from mistakes, as well as being an ongoing process of professional development.

“… over a series of 27,000 patients or so you develop an intuition and you develop some strategy. You’ve made plenty of mistakes so you’ve learnt over time.” [E18].

“We’re taught it as medical students - we learnt it more as residents and registrars. It’s just an accumulative [process] of learning how to take a history and direct the history.” [E8].

Students also referred to the value of practice, but it was clear that not all practice opportunities were equal, with several students contrasting the value of role-plays with simulated patients (actors) to those with peers (playing patient roles).

“I don’t think it’s that great interviewing other medical students. I’m not sure that it’s that beneficial. You’re interviewing people that know exactly what you want or you’re getting at, sometimes their answers are artificial and tailored and fake.” [S5].

“Having two students run a mock interview is a lot more difficult than having an actor … if it’s an actor it’s far more real.” [S12].

“… with friends, we all know sort of how it’s meant to be and so the answers they give might not be like how a patient would normally give it…” [S8].

“When you’re in the hot seat it’s quite challenging to come up with the right questions and get the patient to deliver the right information. [Actors] are good, they do play their roles, so they don’t sit there and spill their story, they dole it out to you based on how relevant the questions are.” [S6].

This concept of the realism of role-plays was reinforced in comments made by two students who indicated that they had a different approach between observed or assessed interactions and those with real patients:

“I guess it depends on the time constraints and who’s watching. If no one else is there I’d probably be more leading, trying to get the information that I know that I need to get on with my job, but if someone else is watching me and for the purpose of assessment I tend to be more open-ended with my questions and allow patients to just talk and tell their story.” [S2].

“I hate mock interviews. I hate doing them because I think you’ve got a lot of pressure on you and you know that your communication skills are being watched; … I come away feeling very frustrated a lot of the time from them, because I suppose I feel like I’m being judged and I don’t think I’m giving maybe a real performance or a realistic one.” [S20].

The discomfort of having many people watching role-plays was also commonly raised, and cited as causing anxiety among students.

“They’re awful, though, when you normally have 10 classmates sitting around and you don’t know what’s happening, and you’re always getting judged as the doctor, so you don’t want to make a mistake. So I found them quite stressful. In the skills lab, we have a Simulation Man … and it’s all videotaped in one room and you can watch it in another room, so it would probably actually be beneficial if people were in another room watching...it’s kind of out of sight, out of mind.” [S17].

The value of practicing in the safety of a simulated environment with actors was also discussed, with students appreciating the opportunities to “pause at different points and … discuss what techniques you’re using” [S15] and practice techniques before using them with real patients.

“We do a little bit of an interview and then it gets stopped and we talk about how we think we’ve went [sic] you can feel like you can try a few different things that you might not otherwise try in front of an actual patient, when you could get an awkward response or muddle your words and stuff things up a little bit. It makes you feel a bit more confident in using those skills.” [S11].

The most highly valued practice opportunities were those with real patients:

“I think any interviewing is better than no interviewing, but whether it translates to patient care, yeah, but not that much …. It’s just so different to when communicating with patients.” [S5].

“I think the only way you can get any better is with real people ….” [S15].

### The value of role-models

#### Positive role-models

Three clinicians and four students described the importance of mentorship on their learning to become effective communicators. Clinicians discussed their role as mentors in training junior staff and their own learning from “watching [their] mentors.” [E1].

“… learn about what sort of a doctor you want to be and who you’re trying to emulate like who are your good role models.” [P13].

Students commented on opportunities to observe consultations, and desires for more such experiences:

“They seem to make it look really easy, and I think they probably use a lot of strategies that I don’t even pick up on.” [S11].

“It would be nice to observe more senior people interact with real live patients.” [S1].

#### The importance of feedback

Several students and clinicians made reference to the importance of feedback in teaching:

“I’ll sit with [a junior doctor] and watch a consultation and afterwards we’ll discuss what he did do, what he didn’t do … in another month’s time I’ll go there again and see if he’s picked it up.” [E11].

[The teaching experience could be improved] “if we had an opportunity to talk to someone, do a role play, and have an assessor write some notes on how we could better handle this.” [S3].

One student struggled with the type of feedback being provided:

“I’ve been trying to incorporate previous feedback which, on the whole, is appalling, the feedback we receive. So you try to incorporate that feedback but each clinician tells you you’re doing wrong, but you’ve changed it from the previous clinician. So there’s a large variability in what is acceptable or appropriate. And that varies day-to-day from a lot of senior clinicians. I don’t think they’re receiving any instruction on how to give feedback.” [S19].

### The structure of a consultation

Five clinicians and nine students discussed the utility of having a solid understanding of effective communication skills which can facilitate gathering information from patients. Clinicians described an ability to have a conversation and then return to the structure to cover outstanding content.

“I think sometimes it might sound to an untrained observer that I’m just having a conversation, and that’s exactly the way I want it. I want it to feel and look that we might be having a conversation, but I’ve got clear ideas what information I want to get … near the end … I might say I’ve missed a few things, I’m just going to ask you now some direct questions to make sure I’ve covered everything.” [E1].

“As much as I hate - like most people - rote learning, I strong [ly] believe you first have to nail the box and dice approach, the textbook.” [E1].

“That’s why you got to rote learn your chosen profession’s questions, so that eventually through that boring phase of your training … it’s all in there, so then it releases you from going ‘what do I say next? What’s the next question I’m meant to ask’.” [E1].

Most students agreed that they had learnt a framework or structure of some kind to guide their history-taking and patient interactions, but the utility of these frameworks was brought into question in their real patient interviews. In particular, the ability to cover both the content of the framework and the varied emotional or social needs of patients poses a challenge to students.

“When you’re inexperienced, sometimes even just the anxiety, confusion can prevent you from displaying empathy, really listening to the patient because you’re so frantic and worried about not missing anything.” [S5].

“I think when you’re new, you’re busy focusing on ‘do I know all the technical guff behind what the presenting symptom means?’ … it’s very hard to ask insightful questions if you can’t discriminate between differential diagnoses, you need to know what’s at the end, so you can ask the right question ...” [S6].

“[Experience helps you] being able to be flexible with the way that you interact with patients, not everyone’s going to respond in the same way to the information presented in one way, it needs to be variable.” [S2].

“The mock interviews didn’t prepare me: they had been given a mock scenario, and it was just sort of back pain or backache, I knew how to respond to those things because I’d been prepared to respond to those things particularly, so I haven’t really interacted with a patient that has had more serious conditions.” [S1].

The clinicians’ reflections also reinforced the challenge for students and junior doctors in balancing these aspects of the consultation and the uncertainty inherent in dealing with a range of complex individuals and issues.

“You can tell they [students and junior doctors] are not listening properly, because the person gives them some juicy information and it’s like they didn’t hear.” [E1].

“I think what junior doctors often do is uncomfortable with the uncertainty of those situations, they’re much happier to have an answer that you should do this, this is what medicine says, as opposed to balancing what medicine says and the patients’ priorities and patients’ thoughts … It’s much easier to have a guideline that’s very definite.” [E3].

### Confidence

Lack of confidence emerged as an important factor throughout each of the themes above, but it also stands alone as a theme, given the capacity for effective education to build confidence in students. Constructive feedback and opportunities to practice can build confidence which can be linked with job or learner satisfaction and ultimately can lead to improved patient outcomes. This topic was raised by four of the experts and four of the students, with key sentiments illustrated in:

“I wish I was even more confident... patients can sense that, and if … they’re more comfortable and more open ... it encourages them to partake in the interview better if they’re trusting you more.” [S5].

“Often the young ones [students] - until they get their confidence up - are just stuck in their little bubble of ‘this is my scope of practice, these are the questions I’ve got to ask’ ….” . [E10].

“I think communication is hard … I’ve been learning a lot about it in the last five years and it’s been opening my world up and it really helps my satisfaction with my work.” [E14].

## Discussion

This qualitative work demonstrates a need for improvement in the teaching of clinical communication skills for medical students. The themes emerging reinforce the challenges identified in previous literature, with the need for feedback and mentoring, the realism and transferability of role-play skills to clinical practice, and the challenge of teaching a structured approach to the medical consultation which inherently requires flexibility. Confidence was a theme running through each of the others, and is an important goal of all education, with both students and clinicians able to improve their confidence through learning and practice. The fact that both very experienced clinicians, and a new generation of future doctors largely agree on the importance of communication skills in the medical consultation is reassuring. The identification of gaps in learning by both groups however, suggests that work is needed to support communication skills training in medical education and the transition to practice.

There was strong consensus among both clinicians and students that experience is likely to be the most valuable tool in developing clinical communication skills. While the clinicians were chosen for their reputation as being good communicators, it is not always the case that experience develops good skills or habits. As Spagnoletti et al. so aptly state, “Experience alone does not reliably improve physicians’ ability to communicate effectively, … which is why nearly every medical school and many residency and fellowship programs include curricula devoted to improving physicians’ communication skills” [41]. Studies have demonstrated that more experienced participants do not improve as much as less experienced participants from interview training (use of open questions). The authors postulated that individuals who are very experienced tend to routinely use specific questions, and that learning a new skill can interfere with their habitual approach [42]. There remains a challenge for medical training to ensure that practice and experience can indeed be valuable in improving skills.

In the absence of the thousands of consultations referred to by experienced clinicians, it seems that role-play with simulated patients, including structured feedback, is highly valued. Consistent with medical education literature supporting the use of experiential learning and simulated patients as providing safe opportunities to practice [24, 25, 27, 43], students valued role-play with simulated patients over that with peers, but did cite a sense of pressure associated with being observed in group settings. Specific teaching approaches have been developed to manage small group environments [44] but medical faculty need to be supported and trained to deliver this teaching effectively. Comments from the experienced clinicians demonstrate that they did not receive formal training in communication skills during their own study, thus are not necessarily well placed to understand or deliver effective teaching in this area.

While short-term outcomes of educational interventions using role-play with simulated patients have been consistently demonstrated, the level of application of acquired skills to actual patient interactions remains unclear. The transfer of classroom-developed skills to clinical settings is a challenge for medical education and is associated with the pressure students’ feel from supervisors in clinical settings [35]. The comments made by students about the realism of their performance in simulation resonates with these concerns, and with previous reports indicating that physicians also perform better in assessments with simulated patients than they do in daily practice [21, 45, 46].

Evidence also emphasises the importance of supervision and feedback in effective training. Inconsistent or absent feedback can be counterproductive and reinforce bad habits [34, 47, 48]. The danger of a lack of feedback could be very real for the students interviewed in this study, with comments regarding inconsistent feedback and a desire to observe and learn from senior clinicians. There is evidence that students’ patient-centredness and empathetic communication declines over time in medical school, with the student-supervisor relationship said to be crucial to the development of these skills [49]. Mirroring patient centredness through student-centred teaching is recommended [49] but was not reflected in the learning experiences of the experienced clinicians or students in this study. Several clinicians mentioned observing senior students or junior doctors and providing tailored feedback to them, but most of the more junior students in this study had not yet experienced such learning opportunities. There is a need for mentors or role-models in clinical settings who can model patient-centred communication approaches and provide feedback to students on their own consultations with patients. In the absence of opportunities for repetitive practice and supervision in clinical settings, involving and training clinicians in classroom teaching could enhance the authenticity of the experience for students [24].

Faculty training is lacking in many medical schools for a range of reasons [43, 50]. Clinicians are often focused on the medical content, and have little or no training in educational approaches, yet they are tasked with supervising students and providing feedback on their communication with patients [34]. While clinicians might have good knowledge, or even good practice of communication skills, this does not mean that by default they have the skills to teach students how to apply various communication skills in appropriate clinical contexts [50]. In many cases, students are not observed performing patient interviews, and when they are, feedback is poor or absent [48].

Another emerging theme was the importance of structure in the medical consultation. The comments made by clinicians were in keeping with Silverman’s stance on structure as a tool to ‘set us free’ [51]. Silverman (2015) describes structure as essential for conceptualising the complex process of clinical communication into manageable elements [51]. Silverman and Kurtz advocate for the use of a frameworks of the medical consultation to facilitate teaching/learning a set of core tasks and skills which act as a foundation upon which to build further skills, and as resource for dealing with more challenging situations [27].

Experienced clinicians echoed Silverman’s description that structure allows for flexibility, with the security of knowing that one can have a conversation but return to the structure to ensure completeness. The students, however talked about the structure as a more rigid guide, and about struggling to achieve this flexibility in their patient encounters and role-plays. Comments from students suggest that for them, with their lack of experience, structure can be a burden, hindering them from engaging fully with the patient. The comment made by one student, that mock-interviews had failed to prepare them for real situations due to the very focused nature of such practice (not covering a broad range of clinical scenarios) emphasises this point.

As described by Kurtz and Silverman (1998), comfort with flexibility is a core goal of medical education: “Communication training should increase rather than reduce flexibility by providing an expanded repertoire of skills that physicians can adeptly and intentionally choose to use as they require” [52]. This is a complex skill however, which is learnt over time, and the development of students’ skills from the early, structured phase to the more flexible and confident later phases of their training remains a challenge. In the early phases of training, students rely on the structure so heavily that they can get away without knowing what it is that they really need to know and why they are asking certain questions. Researchers at one US medical school have reported on the use of applied improvisation to develop students’ skills in communication, collaboration, and adaptability [53]. This approach takes principles proven to be effective in performance settings and applies them to interactions with patients. Development of such skills may be one way to help students more rapidly reach comfort with flexibility.

In the investigative interviewing field, innovative approaches to training have focused on teaching core questioning habits until these are learnt to a high standard, before giving them practice in various questioning scenarios [54, 55]. This training involves identifying question types through a multitude of interactive exercises as well as rote learning of effective question stems so that they are at the ‘tip of the tongue’ [55]. Trainees must demonstrate proficiency in identifying and producing effective questions in advance of putting the skills into practice in mock interviews. The reason for structuring training in this manner is that core communication skills underlie interviewing in most situations and populations [56].

The challenge of teaching students to communicate flexibly parallels that of the integration of ‘process’ skills with the content of the medical consultation which was described by Kurtz et al. in 2003 and remains a challenge in medical education. Some dilemmas identified were the support and teaching of communication beyond the formal classroom curriculum, the continuation of communication training into clinical placements and residency, and the application of communication skills in real-life at a professional level of competence [24]. The themes emerging from the current study suggest that these dilemmas remain relevant today, with medical students indicating that this integration of process and content, and the support of clinical faculty as teachers and mentors has not been fully achieved.

While the recommendations for medical education that emerge from this study are not new, it seems that the approaches accepted as best practice in teaching clinical communication skills are not being uniformly implemented. It is well established that small group practice with simulated patients and structured feedback is ideal. The students in this study had mostly experienced both peer and simulated-patient role-play and indicated that the value of working with simulated patients far exceeded that of peers. Such observations have also been made in the investigative interviewing field. Powell et al. (2008) found that child protection workers who engaged in mock interviews with trained actors had better post-training outcomes than those who completed mock interviews with peers. Actors were not trained to behave childlike, but rather to respond to questions in a way that mimicked the response styles in the field that typically precipitate a deviation from good questions [57].

Despite clear benefits of trained actors for role-plays, there are barriers to implementing this type of training widely and routinely, including cost and clinician engagement in teaching and faculty development. Powell and colleagues (2008) developed training manuals that provide clear instruction on how to play a vulnerable respondent in mock interviews, and are currently testing the ease – or difficulty – with which others can adopt the guidelines. This procedure could be adopted in medical education, such that senior students or teaching assistants could play the role of a patient in a way that shapes learning. Computer-generated avatars are also used in medical education and investigative interviewing training to provide standardised and cost-effective learning experiences [55, 58]. Although the initial cost to develop avatars may be high, they can be used at convenient times for learners and can provide immediate feedback; important criteria for effective learning [54].

### Limitations

This study is limited in its inclusion of a group of students in different year levels of a single graduate entry medical program. The experience of medical students in other programs, or different years of study, may be different. Also, as is reflected in the high average age and inclusion of very mature age students, the participating students are likely to represent a very keen and motivated group, not necessarily representative of the whole cohort. Further, the experienced clinicians in this study were recruited through a mixture of convenience and snowball sampling, using recommendations of personal contacts who verified that the participants were highly experienced clinicians and good communicators. The corroboration between students and clinicians in the identified themes, however, lends weight to the validity of this data.

## Conclusions

The current study provides a picture of the current state of communication skills training in medical education, and an opportunity for educators to consider the next frontiers of this training. While there is evidence that past and present approaches have improved the teaching of medical communication skills,there still remains a long way to go towards implementing best practice medical interviewing and achieving long-term goals in clinician behaviour. The key ingredients of role-play practice, feedback, and clinical supervision are well accepted but the quality of each of these ingredients can vary and is critical to their success. One key need identified for medical education is to focus on the development of skills to help students better cope with the need for flexibility in communication. The second key need identified here is the identification of positive role-models in clinical practice to demonstrate what students can aspire to, and to share the value of their experience with students. There is scope for medical education to adopt innovative interviewing approaches and to learn from professionals who conduct investigative interviewing in other fields.

## Supplementary information

**Additional file 1.** Qualitative Interview Protocol. Information Gathering in Medicine – Qualitative Interview Protocol.

## Data Availability

The datasets generated and analysed during the current study are not publicly available due to the fact that with small numbers and the sharing of personal experiences, participants could be potentially identifiable. Data is available from the corresponding author on reasonable request.
