# Supplementary material for: Assessing the training needs of medical students in patient information gathering
Source: BMC Med Educ. 2020 Mar 2;20:61. doi: 10.1186/s12909-020-1975-2 (PMC7053046; doi:10.1186/s12909-020-1975-2)
Supplement: Supplementary file 1 — Additional file 1. Qualitative Interview Protocol. Information Gathering in Medicine – Qualitative Interview Protocol. [file 12909_2020_1975_MOESM1_ESM.docx]

Additional File

**Information Gathering in Medicine – Qualitative Interview Protocol**

Part 1: Demographic Information

*Collect participant age, gender identity, year of birth, first language, year of medical school or years of experience practicing medicine and ask what kind of communication skills training they have had.*

Part 2: Interviewing approach

*All questions can be followed up with requests for elaboration (e.g., “Tell me more about that”) as appropriate.*

1. Do you use any particular protocol or proforma when communicating with patients?

2. Regarding communication, are there any challenging situations you face/have faced when conducting a session with patients?

3. What are your strengths and weaknesses communicating with patients?

4. Can you describe what makes an ideal communicator?
